# Health service improvement using positive patient feedback: Systematic scoping review

**DOI:** 10.1371/journal.pone.0275045

**Published:** 2023-10-05

**Authors:** Rebecca Lloyd, James Munro, Kerry Evans, Amy Gaskin-Williams, Ada Hui, Mark Pearson, Mike Slade, Yasuhiro Kotera, Giskin Day, Joanne Loughlin-Ridley, Clare Enston, Stefan Rennick-Egglestone

**Affiliations:** 1 School of Health Sciences, Institute of Mental Health, University of Nottingham, Nottingham, United Kingdom; 2 Care Opinion, Sheffield, United Kingdom; 3 School of Health Sciences, University of Nottingham, Nottingham, United Kingdom; 4 Nottinghamshire Healthcare NHS Foundation Trust, Nottingham, United Kingdom; 5 Health and Community Participation Division, Faculty of Nursing and Health Sciences, Nord University, Namsos, Norway; 6 Nightingale Faculty of Nursing, Midwifery and Palliative Care, King’s College London, London, United Kingdom; 7 Faculty of Medicine, Imperial College London, London, United Kingdom; University of Sharjah, UNITED ARAB EMIRATES

## Abstract

**Background:**

Healthcare services regularly receive patient feedback, most of which is positive. Empirical studies suggest that health services can use positive feedback to create patient benefit. Our aim was to map all available empirical evidence for how positive patient feedback creates change in healthcare settings.

**Methods:**

Empirical studies in English were systematically identified through database searches (ACM Digital Library, AMED, ASSIA, CINAHL, MEDLINE and PsycINFO), forwards and backwards citation, and expert consultation. We summarise the characteristics of included studies and the feedback they consider, present a thematic synthesis of qualitative findings, and provide narrative summaries of quantitative findings.

**Results:**

68 papers were included, describing research conducted across six continents, with qualitative (n = 51), quantitative (n = 10), and mixed (n = 7) methods. Only two studies were interventional. The most common settings were hospitals (n = 27) and community healthcare (n = 19). The most common recipients were nurses (n = 29). Most outcomes described were desirable. These were categorised as (a) short-term emotional change for healthcare workers (including feeling motivated and improved psychological wellbeing); (b) work-home interactional change for healthcare workers (such as improved home-life relationships); (c) work-related change for healthcare workers (such as improved performance and staff retention). Some undesirable outcomes were described, including envy when not receiving positive feedback. The impact of feedback may be moderated by characteristics of particular healthcare roles, such as night shift workers having less interaction time with patients. Some factors moderating the change created by feedback are modifiable.

**Conclusion:**

Further interventional research is required to assess the effectiveness and cost-effectiveness of receiving positive feedback in creating specific forms of change such as increases in staff retention. Healthcare managers may wish to use positive feedback more regularly, and to address barriers to staff receiving feedback.

## Introduction

Health service staff regularly receive feedback about the treatment provided to patients, including from the patients themselves, and from family members, and informal carers [1]. Whilst some feedback is solicited through local or national surveys [2, 3], the most frequent form of patient feedback is unsolicited informal feedback [4], which can be exchanged through conversations day-to-day [1], but can also be received via letters to healthcare staff, and posts on online forums [5]. Some patient feedback is used in continuous professional development for healthcare staff. For example, the UK General Medical Council (GMC) require reflection on feedback from service users at least once in each five year revalidation cycle [6]. Service users may want to give feedback to acknowledge, reward, and promote desired behaviour in healthcare staff [7]. Patient feedback differs from patient engagement, which refers to patients taking an active role in their healthcare experience to meet personal objectives such as accessing additional support groups [8].

Patient feedback is given in abundance, and can be used to create meaningful change within healthcare services [5]. In one case study, accounts of distress during admission to mental health inpatient services were used as a resource to inform efforts to redesign services. An 80% drop in complaints was observed over the following 14 months after implementation [9]. In England, the Care Quality Commission has demonstrated that the aggregation of very recent feedback can be used to identify in near real time high-risk priorities for inspection, enabling the management of a declining budget [10]. The Friends and Family Test, a solicited online survey, has been used to monitor the implementation of remote (e.g. video and telephone) appointments during the COVID-19 pandemic, including through identifying positive perceptions of online appointment such as reduced ecological impact [11]. A review by the National Institute for Health Research has recommended that healthcare organisations embrace all forms of feedback as an opportunity to review care [12].

There are a range of organisational barriers to the effective use of patient feedback by health services [13]. Staff can lack the time or skills required to interpret formal feedback [5], and might be reluctant to engage with feedback communicated informally through online platforms such as Facebook or Twitter [14, 15]. In some contexts, online feedback is emerging at a faster rate than health services can respond to [12]. An example is Care Opinion, an online service for the collection of feedback that enables staff responses. A case study evaluation has concluded that conversations are often closed with a ‘thank you’ in response to positive feedback rather than with an account of how this feedback was used to create change [5]. Even where informal feedback is acted on by healthcare staff, the improvements made are often informally implemented in real-time and hence are not captured by quality improvement methods [16]. In a realist evaluation of the use of patient feedback in medical revalidation, concerns were expressed that medical defensiveness, grounded in historical power differences between clinician and patient and an assumed lack of knowledge on the part of the patient, may limit the change that can be created by patient feedback [17].

Healthcare staff may assume that feedback is negative in tone [18], and can dismiss or fail to value positive feedback [14]. However, positive feedback is much more common than negative feedback. For example, a computer-assisted linguistic analysis of 228,113 comments posted on the UK’s National Health Service (NHS) Choices website found positive evaluations to be three times as likely as negative [19]. Positive feedback is evident in a variety of forms, such as favourable responses to surveys [5], online comments [14], compliment letters [7], and informal thanks [5] and may be conceptualised as including material displays, such as gift-giving, cards, and donations to healthcare services [20]. When presented in written form, positive feedback tends to be shorter, often expressed just as a single word such as ‘fantastic’ [14]. Positive and negative evaluations may also be given in combination, forming ‘mixed’ feedback [21]. Increasingly, feedback is received through online sources; a multi-method programme of 5 studies (the INQUIRE study) concluded that online feedback is mostly positive in tone [18].

### Expressions of gratitude to healthcare staff

Gratitude can be conceptualised as the communication of an emotion or state which signals recognition that others have done something to benefit us often for the purpose of reciprocating for the other’s actions [22]. In some cases, expressions of gratitude can serve as a positive evaluation of an individual or group accomplishment, and hence might be thought of as a form of positive feedback. For example, grateful postcards and letters sent to palliative care units from patients and families recognised the care and treatment received, the value of palliative care, and offered messages of support and encouragement about the service [23]. Similarly, throughout the COVID-19 pandemic, many healthcare service users used Twitter to express their gratitude for the work, effort, saving and caring of healthcare staff and services [24] and in Japan, healthcare workers reported that positive communication and acknowledgement, including from patients, acted as a mental health resource [25].

However, not all expressions of gratitude will be given with the intention of recognising accomplishments. Some patients habitually thank healthcare staff in the expectation of ensuring continuation of good treatment [26]. Similarly, not all positive feedback will include expressions of gratitude, with some offering objective descriptions of excellent care and treatment practices. The current review positions expressions of gratitude towards healthcare staff as a potential form of positive feedback, acknowledging how these concepts interrelate and discriminating between them where possible.

Three reviews have investigated the value of gratitude in healthcare settings [27–29]. A meta-narrative review of 56 studies investigated gratitude in healthcare with a particular focus of interpersonal experiences [28]. The review described how gratitude can act as ‘social capital’ as it empowers and motivates recipients through strengthened social bonds, connectedness, and an increased willingness to reciprocate. Day (2020) also highlights how patient gratitude can benefit staff wellbeing, such as being protective against burnout and having physical health benefits and may be an indicator of quality of care. A scoping review [27] included 32 studies from three databases, and examined the characteristics, focus, and effects of gratitude. It found that gratitude influenced healthcare professionals professionally and personally, generating positive feelings such as pride, satisfaction, and a sense of wellbeing. It also generated reciprocal gratitude among other healthcare professionals. The review highlighted a limited evidence base and concluded that a systematic investigation into the effects of patient gratitude was needed [30].

A narrower systematised review which synthesised evidence on the impact of gratitude in healthcare settings included 23 studies from three databases [29]. The review found one harmful change, where service user gift-giving resulted in healthcare staff feeling tension and pressure to meet patient expectations, undermining the service user-professional relationship. The review found that patient gratitude can also create helpful changes for healthcare staff, identified as work-related change (such as improved team performance and work-related satisfaction), direct benefits to staff health (such as increased sleep quality and decreased headaches), and proximal emotional change (such as feeling rewarded, proud, motivated, and fulfilled). In some cases, change was mediated by team information sharing, and was moderated by the psychological demands of the job role. No meta-analysis work was conducted, and hence the review did not provide evidence on the effectiveness of gratitude in creating change.

### Aims and objectives

Prior studies suggest that positive patient feedback can create change in health services that benefits patients. It is possible that positive feedback might be more effective than negative feedback at creating change. For example, positive feedback might enable the identification of specific good practices for replication elsewhere. However, we are not aware of any review that has systematically assessed the empirical evidence on health service change through positive patient feedback, and hence the current state of knowledge is uncertain.

For this paper, our aim is to map all available empirical evidence for how positive patient feedback received by health services about care and treatment can create change within healthcare settings. The objectives are (1) to describe the characteristics of all existing research studies; (2) to describe the characteristics of positive patient feedback considered in these studies; (3) to identify measures used to quantify change due to positive patient feedback; (4) to describe types of change and how it occurs; (5) to identify priorities for research; and (6) (where possible given the current evidence) to make recommendations for health service use.

## Methods

We had originally intended to conduct a systematic review of all available empirical research studies, and hence we prospectively registered a systematic review protocol with the Open Science Framework (https://osf.io/5x46c). We identified our included papers in accordance with this protocol. However, on inspection, we found that the forms of change described in these papers were broad and heterogeneous, with very few interventional studies. Hence, we adopted an aim of mapping this evidence, so as to provide an overview of the current state of evidence in this field, and hence to guide research future work. Where relevant to a systematic scoping review, the 2021 update of the Preferred Reporting Items for Systematic Reviews and Meta-Analyses (PRISMA) checklist was used to structure reporting as originally planned [31], but we also checked our reporting against established guidance for conducting systematic scoping reviews [32].

### Search strategy

#### Electronic database searches

Databases were selected to cover a range of domains relating to healthcare service delivery. Searches were conducted from inception to 18^th^ March 2022 on PsycINFO, AMED, MEDLINE, CINAHL, and the ACM Digital Library (ACM DL), and from inception to 15^th^ December 2021 on ASSIA (the shorter date was due to a constraint in institutional access). The ACM DL indexes papers where computation and human interaction with technology is a primary focus and was included as feedback is frequently collected via electronic systems.

Search terminology was extensively tested during a previously conducted systematised review focusing on expressions of patient gratitude [29], extended for the current review to encompass positive feedback beyond gratitude and healthcare systems more generally, and informed by the learning from the scoping searches. Scoping searches identified terms which were synonymous with ‘positive feedback’, such as ‘positive evaluation’ and ‘praise’, and terms which described healthcare systems, such as ‘healthcare services’ and ‘healthcare communities’.

Search terms which linked less closely to positive feedback but produced a high volume of documents, such as recognition, were searched in titles only. In the initial filter by title, the screening team took care not to exclude papers in the event of ambiguity.

The following search strategy was used for MEDLINE, PsycINFO, and AMED (all searched through Ovid):

1. Health* staff.ti,ab.
2. Health* worker*.ti,ab.
3. Medical staff.ti,ab.
4. Medical worker*.ti,ab.
5. Exp Health Personnel/
6. Health* system*.ti,ab.
7. Health* service*.ti,ab.
8. Health* organi#ation*.ti,ab.
9. Health* communit*.ti,ab.
10. 1 or 2 or 3 or 4 or 5 or 6 or 7 or 8 or 9
11. Grat*.ti,ab.
12. Appreciat*.ti,ab.
13. Recog*.ti.
14. Thank*.ti.
15. Positive* feedback.ti,ab.
16. Positive* evaluat*.ti,ab.
17. Praise*.ti,ab.
18. 11 or 12 or 13 or 14 or 15 or 16 or 17
19. 10 and 18
20. Remove duplicates from 19

This search strategy was amended for CINAHL and ASSIA (amendments in S1 File).

The ACM Digital Library only allows searches constructed using combinations of keywords, which generates a series of online pages of possible matches in order of relevance. Keyword combinations were identified from the MEDLINE search strategy (searches in S1 File). For each keyword combination, results pages were sequentially inspected for potentially includable documents, and inspection was discontinued when three subsequent pages of non-relevant results were observed.

When developing the search strategy, documents from the prior review [29] were used as marker papers to evaluate search strategy sensitivity.

#### Citation tracking

Reference lists for included documents were manually inspected for further includable documents (backwards referencing). Forward referencing of included documents was conducted using Google Scholar. Forward and backward citation was repeated on additional included documents until no further documents were included.

#### Expert consultation

Once the final list of includable documents from electronic databases was identified, three experts in healthcare service delivery were asked to identify any potentially includable documents which had been omitted. Experts consisted of a healthcare manager responsible for feedback, an academic expert, and a technology creator who collects feedback about healthcare. Proposed documents were inspected for inclusion by the researcher. Forwards and backward referencing was conducted on additional included documents identified during expert consultation and repeated until no further documents were included.

### Document inclusion

The Population, Intervention, Comparison, Outcome, Study Design (PICOS) search tool was used to specify inclusion [33].

#### Study design

We included any empirical study where the full text is publicly available in English, with a clearly defined research method. Documents were included which described change that occurred within healthcare services that was attributed within the document to positive patient feedback.

Documents describing systematic, literature, or scoping reviews, policy statements, conference abstracts, protocols, and documents presented in a blog format were excluded. Documents were excluded where it was unclear whether change occurred as a result of positive feedback, where the identified change preceded positive feedback or directionality was ambiguous (e.g., where a change in healthcare staff or systems caused positive service user feedback), or where the impact of positive feedback was not presented as a study finding but was briefly mentioned as a discussion point.

#### Context

Included documents described research in the context of a healthcare setting, defined as any formal service where healthcare is being delivered, such as in hospitals, outpatient services, hospices, healthcare education, or correctional medical facilities. This was not limited to private or public healthcare services. Documents describing community healthcare settings were also included if staff were providing a formal healthcare service in the community. Documents were excluded where they describe positive feedback occurring within a healthcare system in relation to research being conducted, such as feedback about participation in a randomized clinical trial.

#### Intervention

Positive patient feedback was defined as a response from healthcare service users, families or the community indicating concordance between desired and actual experiences regarding care or treatment, delivered to healthcare staff or systems. Included documents described the voluntary expression of positive feedback from healthcare service users, their families, or community members, relating to the care or treatment provided, with healthcare workers or healthcare services as recipients. This included positive feedback expressed verbally and in invariant forms (such as in writing), and positive feedback provided both in-person and remotely (such as online). Expressions of gratitude were included as they may indicate service user feelings about care and treatment and hence can be used as a source of information by healthcare staff or systems. Studies describing ‘recognition’ of healthcare staff or services in relation to appreciation of care and treatment provided were included.

Documents were excluded if (1) the type of service user feedback was not identified as positive, was negative or mixed, ambiguous, or was hypothetical (2) the source of positive feedback was not healthcare service users, families, communities, or was ambiguous (3) positive feedback from healthcare service users, families, or communities was not distinct from feedback provided by peers or the organisation, or (4) expressions of positive feedback were not voluntary (for example, where service users felt that their care and treatment may be negatively impacted if they do not express positive feedback). Feedback was assumed to be given voluntarily unless otherwise stated. Documents describing recognition awards or honours informed by the treatment and care experiences of healthcare service users, such as the Diseases Attacking the Immune System (DAISY) Award [34], were excluded. Similarly, documents describing feedback given via Appreciative Inquiry (a strength-based approach to creating change with a focus on appreciation and positive conversations) were excluded if service user involvement was not explicitly stated or distinguishable from peer or organizational feedback [35]. Documents describing donations or gifts to healthcare services were excluded if the motivation for donation was not explicitly described as positive feedback or gratitude towards the healthcare staff or system [29]. Studies which describe positive recognition of healthcare staff regarding social status rather than care or treatment provided, such as community support, approval, acceptance, or respect, were excluded [36]. Studies were also excluded where healthcare service user satisfaction with care and treatment was described, but not explicitly delivered as positive feedback to healthcare staff or services.

#### Participants

Included documents described participants as working within a formal healthcare environment. The following were in scope: paid or volunteer workers within any healthcare system worldwide; students carrying out a formal healthcare role as part of their studies. Documents describing research into healthcare systems at an organizational level (e.g., where there were no staff participants) were also included. Healthcare systems were defined as any healthcare structure delivering care services to healthcare users.

Documents were excluded where authors did not state whether feedback was provided within a healthcare setting, if participant roles were informal such as unpaid familial caregivers, or if participants were unable to receive feedback.

#### Outcome

Change was in scope if it related to individual healthcare staff (such as behavioural, emotional, and attitudinal shifts), or to systematic or procedural change within healthcare structures.

### Document selection and data abstraction

Documents from database searches were exported to EndNote [37] and duplicates were removed. Documents were screened for eligibility, filtered on title in stage one and abstract in stage two. Concordance checking was conducted on a randomly selected 20% of exclusions by a second researcher [SRE] for both stages (title and abstract) of exclusion. Selection processes were piloted until a concordance rate of 95% was achieved on exclusions. Stage 3 screened remaining documents for eligibility based on full text. Retrieved documents were reviewed for inclusion by two researchers, with 100% concordance required on inclusions and exclusions for Stage 3. Uncertainty about the eligibility of a document from both researchers led to it being carried forward to the next stage of screening. At Stage 3, reasons for exclusion were recorded and agreement was required between RL and SRE.

#### Data abstraction

A data abstraction table (DAT) was amended from the systematised review [29] and piloted using a small number of includable documents to ensure appropriate and efficient design.

Understanding the change created by positive patient feedback requires an understanding of the context in which it was given. As such, the DAT included information about country of study, healthcare setting, the type of positive feedback considered, the healthcare role of the feedback recipient, and the status of the person providing feedback (e.g. whether they were a patient, family member, or community member). The DAT also included information on study methodology (such as measures and purpose of measures), and the change observed. For types of feedback, donations were recorded under the higher category of ’gifts’.

Information on change described in included papers was recorded in the DAT. In keeping with prior work on change modelling [38, 39], the observed change was categorised into DAT columns presenting: outcomes, mechanisms, moderators, facilitators, barriers, and mediators. Definitions were drawn from a study which produced a change model through the qualitative analysis of interview transcripts [40]. Outcomes were defined as observed changes that have occurred following positive feedback. Mechanisms were defined as processes which produce change. Moderators were defined as factors which alter the degree of change following positive feedback. Facilitators were defined as factors enhancing the observed change. Barriers were defined as factors impeding the observed change. Mediators were defined as factors creating an indirect pathway between two variables enabling change to occur. When change was described in the DAT, it closely followed the language of the included paper.

Specific links between outcomes, mechanisms, mediators, moderators, facilitators, and barriers were retained in the DAT, for example if an included document presented evidence that a specific outcome was produced by a specific mechanism. Items were listed in all relevant categories where there was variation in categorisation among studies. With the definition above, facilitators and barriers are both specific forms of moderators. These three entities were included to reflect how change was described in included papers. Where papers reported more than one study within a single paper, only data from relevant studies were extracted. The quality of included documents was assessed using the Mixed Methods Appraisal Tool (MMAT) [41] and scores were included in the DAT. If a section of the DAT was not clearly stated in a document, it was recorded as ‘N/A’.

### Data synthesis

Summary tables were produced to describe characteristics of included studies (objective 1), and brief narrative descriptions were produced for papers describing interventional work. Summary tables were produced to identify characteristics of positive patient feedback (objective 2), to identify measures used to quantify change (objective 3), and to identify change (objective 4). For objective 4, moderators, facilitators, and barriers were first combined into two tables reflecting factors that enhance change and factors that hinder change.

For all tables, included items were assessed for similarity. Where items were identified as representing the same underlying construct they were combined, but the review team had an orientation towards not combining items unless necessary so as not to lose information. All remaining items were examined, and grouped into higher level constructs where these were informative.

Tabulated items and higher level constructed were then reviewed by an expert panel consisting of national and local health service representatives experienced with working with patient feedback to create operational change, the director of a company providing a public online feedback platform (JM), and three experienced researchers. Names were revised for clarity and health service relevance.

As a robustness check, change described in papers not meeting a pre-planned quality threshold of 60% was examined. The expert panel recommended an unplanned subgroup analysis comparing change described in mainly public versus mainly private healthcare settings.

For objectives 5 and 6, the expert panel produced initial recommendations, which were reviewed and revised by all authors.

### Reflexive statement

Work in this paper originated in discussions between SRE, AGW and JM. Through these discussions, SRE developed a belief that statutory health services can learn more from experiences of treatment that are positive rather than negative, and that patient feedback might provide a route to accessing information about positive experiences. This position was then initially explored through an MSc research project by RL on health service change created through expressions of patient gratitude (supervisor: SRE), which has been extended by the current funded review. The selected approach to synthesising knowledge on change was influenced by prior change modelling work led by SRE [38, 40], which has been beneficial in enabling intervention development work in a substantial research programme [42], and which in turn was informed by prior work by others [39]. Arguably, this approach to synthesizing knowledge has a bias towards future intervention development work, potentially leading to the selection of concepts which are generative [43], in that they can seed new ideas for interventions.

## Results

### Review process

**Fig 1:**
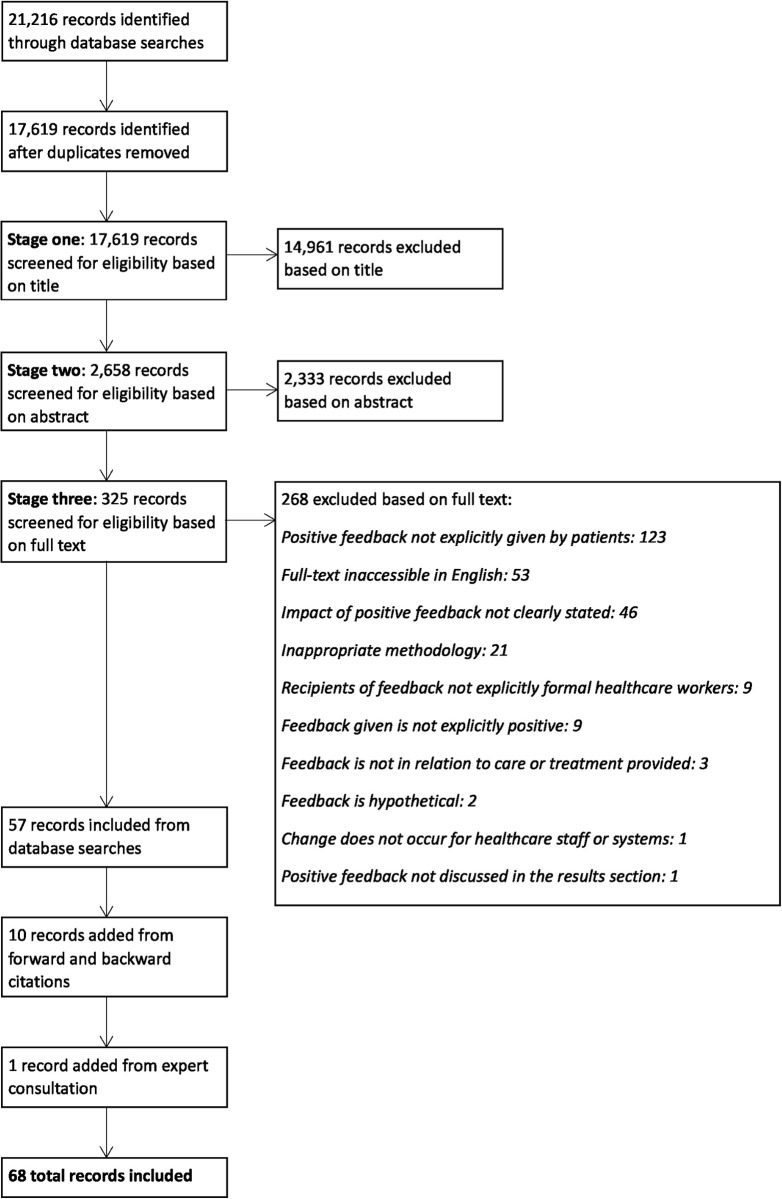
PRISMA flow diagram of included and excluded screening process.

Database searches identified 17,619 records once duplicates were removed. Sixty-eight papers were included (see Fig 1). The PRISMA checklist is in S2 File.

### Objective 1—Characteristics of included studies

A summary DAT is in Table 1, and the full DAT is in S1 Table. One included study presented methodology and results across two papers [44, 45] which were merged to form one record [ID 67]. Where several papers were created from a single study, these were considered companion papers. Three studies had corresponding companion papers [ID 3 and 4; ID 11 and 12; ID 45 and 46].

**Table 1:** Summary data abstraction table.

ID	Reference	Year	Country	Study type	Design	Setting	Sample size
1	Akintola, O. (2010). Perceptions of rewards among volunteer caregivers of people living with AIDS working in faith-based organizations in South Africa: a qualitative study. Journal of the International AIDS Society, 13(1), 1–10. https://doi.org/10.1186/1758-2652-13-22	2010	South Africa	Qualitative (Interviews)	Observational	Community	55
2	Akintola, O., & Chikoko, G. (2016). Factors influencing motivation and job satisfaction among supervisors of community health workers in marginalized communities in South Africa. Human Resources for Health, 14(1), 1–15. https://doi.org/10.1186/s12960-016-0151-6	2016	South Africa	Qualitative (Interviews)	Observational	Community	26
3	Alam, K., & Oliveras, E. (2014). Retention of female volunteer community health workers in Dhaka urban slums: a prospective cohort study. Human Resources for Health, 12(1), 1–11. https://doi.org/10.1186/1478-4491-12-29	2014	Bangladesh	Mixed (Interviews Survey)	Observational	Community	542
4	Alam, K., et al. (2012). Performance of female volunteer community health workers in Dhaka urban slums. Social Science & Medicine, 75(3), 511–515. https://doi.org/10.1016/j.socscimed.2012.03.039	2012	Bangladesh	Mixed (Questionnaire Focus groups Interviews)	Observational	Community	542
5	Alibhai, A. A. (2013). The effectiveness of a volunteer community health worker program to support an antiretroviral treatment program for AIDS patients in western Uganda. Available from ProQuest Dissertations & Theses A&I. http://nottingham.idm.oclc.org/login?url=https://www.proquest.com/dissertations-theses/effectiveness-volunteer-community-health-worker/docview/1504615762/se-2?accountid=8018	2013	Uganda	Mixed (Questionnaire Interviews Focus groups)	Observational	Community	169
6	Aparicio, M., et al. (2019). Gratitude from patients and relatives in palliative care—characteristics and impact: a national survey. BMJ Supportive & Palliative Care. 10.1136/bmjspcare-2019-001858	2019	Spain	Quantitative (Survey)	Observational	Palliative care units Community	186
7	Ashley, C., et al. (2021). The psychological well‐being of primary healthcare nurses during COVID‐19: a qualitative study. Journal of Advanced Nursing, 77(9), 3820–3828. https://doi.org/10.1111/jan.14937	2021	Australia	Qualitative (Interviews)	Observational	GPs Community	25
8	Bakker, D., et al. (2010). Canadian cancer nurses’ views on recruitment and retention. Journal of Nursing Management, 18(2), 205–214. https://doi.org/10.1111/j.1365-2834.2009.01029.x	2010	Canada	Qualitative (Focus groups)	Observational	Oncology Ambulatory care Hospitals Community	91
9	Barnes, A. L. (2015). Relationship between job satisfaction among frontline staff and patient satisfaction: Evidence from community health centers in South Carolina (Doctoral dissertation, University of South Carolina). https://www.proquest.com/docview/1765406972?pq-origsite=gscholar&fromopenview=true	2015	USA	Quantitative (Survey)	Observational	Community	303
10	Beate, A., & Jacobsen, F. F. (2020). The art of caring in selected Norwegian nursing homes: a qualitative approach. International Journal of Caring Sciences, 13(2), 820. https://hdl.handle.net/11250/2738332	2020	Norway	Qualitative (Interviews)	Observational	Nursing homes	11
11	Bhatnagar, A. (2014). Determinants of motivation and job satisfaction among primary health workers: case studies from Nigeria and India (Doctoral dissertation, Johns Hopkins University). http://jhir.library.jhu.edu/handle/1774.2/37851	2014	Nigeria India	Mixed (Interviews Survey)	Observational	Primary health care	29
12	Bhatnagar, A., et al. (2017). Primary health care workers’ views of motivating factors at individual, community and organizational levels: a qualitative study from Nasarawa and Ondo states, Nigeria. The International Journal of Health Planning and Management, 32(2), 217–233. https://doi.org/10.1002/hpm.2342	2017	Nigeria	Qualitative (Interviews)	Observational	Community	29
13	Blank, F. S., et al. (2014). A comparison of patient and nurse expectations regarding nursing care in the emergency department. Journal of Emergency Nursing, 40(4), 317–322. https://doi.org/10.1016/j.jen.2013.02.010	2014	N/A	Mixed (Survey)	Observational	Emergency department	100
14	Cameron, P. J., et al. (2010). Physician retention in rural Alberta: key community factors. Canadian Journal of Public Health, 101(1), 79–82. https://doi.org/10.1007/BF03405568	2010	Canada	Qualitative (Interviews Document review Observations)	Observational	Community	15
15	Chou, W. C., et al. (2006). Perceptions of physicians on the barriers and facilitators to integrating fall risk evaluation and management into practice. Journal of General Internal Medicine, 21(2), 117–122. https://doi.org/10.1007/s11606-006-0244-3	2006	USA	Qualitative (Interviews)	Observational	Primary care offices	18
16	Christiansen, B. (2008). Good work–how is it recognised by the nurse? Journal of Clinical Nursing, 17(12), 1645–1651. https://doi.org/10.1111/j.1365-2702.2007.02139.x	2008	Norway	Qualitative (Interviews)	Observational	Hospitals Clinic	10
17	Ciocănel, A., et al. (2018). Helping, mediating, and gaining recognition: the everyday identity work of Romanian health social workers. Social Work in Health Care, 57(3), 206–219. https://doi.org/10.1080/00981389.2018.1426674	2018	Romania	Qualitative (Interviews)	Observational	Hospitals Emergency department Maternity unit School-based Community Hospice	21
18	Cleary, M., et al. Mental health nurses’ perceptions of good work in an acute setting. International Journal of Mental Health Nursing, 21(5), 471–479. https://doi.org/10.1111/j.1447-0349.2011.00810.x	2012	Australia	Qualitative (Interviews)	Observational	Mental health centres	40
19	Converso, D., et al. (2015). Do positive relations with patients play a protective role for healthcare employees? Effects of patients’ gratitude and support on nurses’ burnout. Frontiers in Psychology, 6, 470. 10.3389/fpsyg.2015.00470	2015	Italy	Quantitative (Questionnaire)	Observational	Hospitals Emergency department Oncology	204
20	Cortese, C. G. (2007). Job satisfaction of Italian nurses: an exploratory study. Journal of Nursing Management, 15(3), 303–312. https://doi.org/10.1111/j.1365-2834.2007.00694.x	2007	Italy	Qualitative (Interviews)	Observational	Hospitals	64
21	Dageid, W., et al. (2016). Sustaining motivation among community health workers in aids care in Kwazulu‐natal, South Africa: challenges and prospects. Journal of Community Psychology, 44(5), 569–585. https://doi.org/10.1002/jcop.21787	2016	South Africa	Qualitative (Interviews)	Observational	Community	12
22	Danet, A. D., et al. (2020). Emotional paths of professional experiences in transplant coordinators. Nefrología (English Edition), 40(1), 75–90. https://doi.org/10.1016/j.nefroe.2019.05.005	2020	Spain	Qualitative (Questionnaire Interviews)	Observational	Hospitals Transplant coordination	22
23	Datiko, D. G., et al. (2015). Exploring providers’ perspectives of a community based TB approach in Southern Ethiopia: implication for community based approaches. BMC Health Services Research, 15(1), 1–9. https://doi.org/10.1186/s12913-015-1149-9	2015	Ethiopia	Qualitative (Interviews)	Observational	Community	37
24	de Oliveira, A. R., et al. (2019). Satisfaction and limitation of primary health care nurses’ work in rural areas. Rural and Remote Health, 19(2), 55–64. https://search.informit.org/doi/10.3316/informit.143753391883465	2019	Brazil	Qualitative (Interviews)	Observational	Family health units	11
25	Fereday, J., & Muir-Cochrane, E. (2006). The role of performance feedback in the self-assessment of competence: a research study with nursing clinicians. Collegian, 13(1), 10–15. https://doi.org/10.1016/S1322-7696(08)60511-9	2006	Australia	Qualitative (Focus groups)	Observational	Hospitals Midwifery General surgical General medical	26
26	Fontanini, R., et al. (2021). Italian nurses’ experiences during the COVID‐19 pandemic: a qualitative analysis of internet posts. International Nursing Review, 68(2), 238–247. https://doi.org/10.1111/inr.12669	2021	Italy	Qualitative (Descriptive study)	Observational	Hospitals Community	380
27	Fort, A. L., & Voltero, L. (2004). Factors affecting the performance of maternal health care providers in Armenia. Human Resources for Health, 2(1), 1–11. https://doi.org/10.1186/1478-4491-2-8	2004	Armenia	Quantitative (Interviews Survey Observations)	Observational	Reproductive health services	285
28	Johansson, M., et al. (2019). Nursing staff’s experiences of intensive care unit diaries: a qualitative study. Nursing in Critical Care, 24(6), 407–413. https://doi.org/10.1111/nicc.12416	2019	Sweden	Qualitative (Focus groups)	Observational	University Hospitals ICU	27
29	Judd, M. J., et al. (2017). Workplace stress, burnout and coping: a qualitative study of the experiences of Australian disability support workers. Health & Social Care in the Community, 25(3), 1109–1117. https://doi.org/10.1111/hsc.12409	2017	Australia	Qualitative (Interviews)	Observational	Disability Services	12
30	Kelly, D., et al. (2020). The experiences of cancer nurses working in four European countries: a qualitative study. European Journal of Oncology Nursing, 49, 101844. https://doi.org/10.1016/j.ejon.2020.101844	2020	Estonia Germany Netherlands UK	Qualitative (Interviews Focus groups)	Observational	Oncology	97
31	Khowaja, K., et al. (2005). Registered nurses perception of work satisfaction at a Tertiary Care University Hospital. Journal of Nursing Management, 13(1), 32–39. https://doi.org/10.1111/j.1365-2834.2004.00507.x	2005	Pakistan	Qualitative (Interviews Focus groups)	Observational	Hospitals Critical care Medical-surgery Ambulatory Maternity Emergency department	45
32	Kim, Y. M., et al. (2008). Factors that enable nurse–patient communication in a family planning context: a positive deviance study. International Journal of Nursing Studies, 45(10), 1411–1421. https://doi.org/10.1016/j.ijnurstu.2008.01.002	2008	Indonesia	Qualitative (Interviews Focus groups)	Observational	Clinic	34
33	MacLeod, M. L., et al. (2021). The meaning of nursing practice for nurses who are retired yet continue to work in a rural or remote community. BMC Nursing, 20(1), 1–13. https://doi.org/10.1186/s12912-021-00721-0	2021	Canada	Qualitative (Survey)	Observational	N/A	101
34	Maharani, C., et al. (2022). The National Health Insurance System of Indonesia and primary care physicians’ job satisfaction: a prospective qualitative study. Family Practice, 39(1), 112–124. https://doi.org/10.1093/fampra/cmab067	2022	Indonesia	Qualitative (Interviews)	Observational	Primary health care	34
35	Martínez-Taboas, A., et al. (2014). Gifts in psychotherapy: attitudes and experiences of Puerto Rican psychotherapists. Revista Puertorriqueña de Psicología, 25(2), 328–339. https://www.redalyc.org/articulo.oa?id=233245622011	2014	Puerto Rico	Quantitative (Questionnaire)	Observational	Private practice Hospitals University	75
36	Minooee, S., et al. (2021). Catastrophic thinking: is it the legacy of traumatic births? Midwives’ experiences of shoulder dystocia complicated births. Women and Birth, 34(1), e38-e46. https://doi.org/10.1016/j.wombi.2020.08.008	2021	Australia	Qualitative (Interviews)	Observational	Hospitals	25
37	Muntz, J., & Dormann, C. (2020). Moderating effects of appreciation on relationships between illegitimate tasks and intrinsic motivation: a two-wave shortitudinal study. European Journal of Work and Organizational Psychology, 29(3), 391–404. https://doi.org/10.1080/1359432X.2019.1706489	2020	Germany	Quantitative (Panel study)	Observational	Hospitals	241
38	Nwala, E. (2015). The impact of nonmonetary job benefits on job retention in rural healthcare (Doctoral dissertation, Capella University). https://www.proquest.com/docview/1735405605?pq-origsite=gscholar&fromopenview=true	2015	USA	Qualitative (Interviews Observations)	Observational	Clinic	13
39	Oluwole, A., et al. (2019). Optimising the performance of frontline implementers engaged in the NTD programme in Nigeria: lessons for strengthening community health systems for universal health coverage. Human Resources for Health, 17(1), 1–16. https://doi.org/10.1186/s12960-019-0419-8	2019	Nigeria	Qualitative (Workshops)	Observational	Community	N/A
40	Ortiz, J. A. (2014). New graduate nurses’ experiences of what accounts for their lack of professional confidence during their first year of practice (Doctoral dissertation, Capella University). https://www.proquest.com/docview/1650654883?pq-origsite=gscholar&fromopenview=true	2014	USA	Qualitative (Interviews)	Observational	Hospitals	12
41	Pal, L. M., et al. (2014). Utilising feedback from patients and their families as a learning strategy in a foundation degree in palliative and supportive care: a qualitative study. Nurse Education Today, 34(3), 319–324. https://doi.org/10.1016/j.nedt.2013.06.012	2014	UK	Qualitative (Focus groups Questionnaire)	Observational	Nursing homes Hospitals Hospices Oncology wards Community	12
42	Pariseault, C. A., et al. (2022). Nurses’ experiences of caring for patients and families during the Covid-19 pandemic: communication challenges. American Journal of Nursing, 122, 22–30. 10.1097/01.NAJ.0000805644.85184.d2	2022	USA	Qualitative (Descriptive study)	Observational	Hospitals	17
43	Peteet, J. R., et al. (1992). Relationships with patients in oncology: can a clinician be a friend? Psychiatry, 55(3), 223–229. https://doi.org/10.1080/00332747.1992.11024596	1992	USA	Mixed (Interviews)	Observational	Oncology	192
44	Pooley, H. M., et al. (2015). The experience of the long-term doctor-patient relationship in consultant nephrenologists. Journal of Renal Care, 41(2), 88–95. https://doi.org/10.1111/jorc.12092	2015	UK	Qualitative (Interviews)	Observational	Renal department	7
45	Prytherch, H., et al. (2012). Maternal and newborn healthcare providers in rural Tanzania: in-depth interviews exploring influences on motivation, performance and job satisfaction. Rural and Remote Health, 12(3), 1–15. https://search.informit.org/doi/10.3316/informit.625974688045681	2012	Tanzania	Qualitative (Interviews)	Observational	Health centres	35
46	Prytherch, H., et al. (2013). Motivation and incentives of rural maternal and neonatal health care providers: a comparison of qualitative findings from Burkina Faso, Ghana and Tanzania. BMC Health Services Research, 13(1), 1–15. https://doi.org/10.1186/1472-6963-13-149	2013	Burkina Faso Ghana Tanzania	Qualitative (Interviews)	Observational	Health centres	35
47	Raingruber, B., & Wolf, T. (2015). Nurse perspectives regarding the meaningfulness of oncology nursing practice. Clinical Journal of Oncology Nursing, 19(3), 292–296. 10.1188/15.CJON.292-296	2015	USA	Qualitative (Interviews)	Observational	Oncology wards Medical-surgical unit	8
48	Reis, M. J. D., et al. (2010). Experiences of nurses in health care for female victims of sexual violence. Revista de Saude Publica, 44, 325–331. https://doi.org/10.1590/S0034-89102010000200013	2010	Brazil	Qualitative (Interviews)	Observational	Sexual violence service	6
49	Riskin, A., et al. (2019). Expressions of gratitude and medical team performance. Pediatrics, 143(4). https://doi.org/10.1542/peds.2018-2043	2019	Israel	Quantitative (Randomised study)	Interventional	Hospitals NICU	172
50	Robinson, D. (2019). Exploring experiences of burnout, engagement, and social support setworks: a qualitative study of hospital medicine physicians (Doctoral dissertation, Colorado State University). https://www.proquest.com/docview/2244361153?pq-origsite=gscholar&fromopenview=true	2019	USA	Mixed (Interviews Survey)	Observational	Hospitals	15
51	Roca, J., et al. (2021). Experiences, emotional responses, and coping skills of nursing students as auxiliary health workers during the peak Covid‐19 pandemic: a qualitative study. International Journal of Mental Health Nursing, 30(5), 1080–1092. https://doi.org/10.1111/inm.12858	2021	Spain	Qualitative (Interviews)	Observational	Nursing homes Hospitals COVID-19 specialized unit	22
52	Ronnie, L. (2019). Intensive care nurses in South Africa: expectations and experiences in a public sector hospital. Journal of Nursing Management, 27(7), 1431–1437. https://doi.org/10.1111/jonm.12826	2019	South Africa	Qualitative (Interviews)	Observational	Hospitals ICU	44
53	Sakai, M., et al. (2013). Home visiting nurses’ attitudes toward caring for dying patients, and related workplace factors. International Journal of Palliative Nursing, 19(4), 195–204. https://doi.org/10.12968/ijpn.2013.19.4.195	2013	Japan	Quantitative (Questionnaire)	Observational	Community	206
54	Seitovirta, J., et al. (2015). Registered nurses’ experiences of rewarding in a Finnish university hospital–an interview study. Journal of Nursing Management, 23(7), 868–878. https://doi.org/10.1111/jonm.12228	2015	Finland	Qualitative (Interviews)	Observational	Hospitals	10
55	Seitovirta, J., et al. (2017). Attention to nurses’ rewarding–an interview study of registered nurses working in primary and private healthcare in Finland. Journal of Clinical Nursing, 26(7–8), 1042–1052. https://doi.org/10.1111/jocn.13459	2017	Finland	Qualitative (Interviews)	Observational	Healthcare organisations	20
56	Smallwood, N., et al. (2021). Moral distress and perceived community views are associated with mental health symptoms in frontline health workers during the COVID-19 pandemic. International Journal of Environmental Research and Public Health, 18(16), 8723. https://doi.org/10.3390/ijerph18168723	2021	Australia	Quantitative (Survey)	Observational	Hospitals	7846
57	Tang, P. M., et al. (2021). How and when service beneficiaries’ gratitude enriches employees’ daily lives. Journal of Applied Psychology, 107(6), 987–1008. https://doi.org/10.1037/apl0000975	2021	China Singapore	Quantitative (Experience Sampling Method)	Observational	Hospitals	275
58	Vachon, M., & Guité-Verret, A. (2020). From powerlessness to recognition the meaning of palliative care clinicians’ experience of suffering. International Journal of Qualitative Studies on Health and Well-being, 15(1), 1852362. https://doi.org/10.1080/17482631.2020.1852362	2020	Canada	Qualitative (Interviews)	Observational	Medical centre	21
59	Vail, L., et al. (2011). Healthcare assistants in general practice: a qualitative study of their experiences. Primary Health Care Research & Development, 12(1), 29–41. https://doi.org/10.1017/S1463423610000204	2011	UK	Qualitative (Interviews)	Observational	GP	14
60	Vandecasteele, T., et al. (2015). Nurses’ perceptions of transgressive behaviour in care relationships: a qualitative study. Journal of Advanced Nursing, 71(12), 2786–2798. https://doi.org/10.1111/jan.12749	2015	Belgium	Qualitative (Interviews)	Observational	Hospitals	18
61	Wahlberg, A. C., & Bjorkman, A. (2018). Expert in nursing care but sometimes disrespected—telenurses’ reflections on their work environment and nursing care. Journal of Clinical Nursing, 27(21–22), 4203–4211. https://doi.org/10.1111/jocn.14622	2018	Sweden	Qualitative (Interviews)	Observational	Telephone service	24
62	Waltz, L. A., et al. (2020). Exploring job satisfaction and workplace engagement in millennial nurses. Journal of Nursing Management, 28(3), 673–681. https://doi.org/10.1111/jonm.12981	2020	USA	Qualitative (Focus groups)	Observational	Hospitals	33
63	Warburton, J., et al. (2014). Extrinsic and intrinsic factors impacting on the retention of older rural healthcare workers in the north Victorian public sector: a qualitative study. Rural and Remote Health, 14(3), 131–146. https://search.informit.org/doi/10.3316/informit.451178784672507	2014	Australia	Qualitative (Interviews)	Observational	N/A	17
64	Wasko, K. (2014). Medical practice in rural Saskatchewan: factors in physician recruitment and retention. Canadian Journal of Rural Medicine, 19(3), 93. https://srpc.ca/resources/Documents/CJRM/vol19n3/pg93.pdf	2014	Canada	Qualitative (Interviews)	Observational	Community	62
65	Weaver, S. H., et al. (2020). The impact of real-time patient feedback using a gamified system. Nursing Management, 51(12), 14–21. 10.1097/01.NUMA.0000721812.13386.81	2020	USA	Mixed (Interviews Focus groups Survey)	Interventional	Hospitals Medical-surgical unit	22
66	Wright, S. M., et al. (2013). Ethical concerns related to grateful patient philanthropy: the physician’s perspective. Journal of General Internal Medicine, 28(5), 645–651. https://doi.org/10.1007/s11606-012-2246-7	2013	USA	Qualitative (Interviews)	Observational	University	20
67	Zulu, J. M., et al. (2015). 1/3. Hope and despair: the community health assistant role in Zambia. British Journal of Healthcare Assistants, 9(9), 458–465. https://doi.org/10.12968/bjha.2015.9.9.458 Zulu, J. M., et al. (2016). Hope and despair 3/3: pluses and minuses for community health assistants in rural Zambia. British Journal of Healthcare Assistants, 10(1), 31–35. https://doi.org/10.12968/bjha.2016.10.1.31	2015 2016	Zambia	Qualitative (Interviews)	Observational	Community	12
68	Zwack, J., & Schweitzer, J. (2013). If every fifth physician is affected by burnout, what about the other four? Resilience strategies of experienced physicians. Academic Medicine, 88(3), 382–389. 10.1097/ACM.0b013e318281696b	2013	Germany	Qualitative (Interviews)	Observational	Hospitals	200

Research was located in 32 countries across six continents (Table 2). Two studies were located in multiple countries [46, 47]. One study did not state the study location [48].

**Table 2:** Research location of included studies in order of quantity. Multiple papers from the same study counted as having a single location unless reporting results from different locations.

Continent	Quantity	Country	Quantity	Study ID(s)
Europe	23	UK	4	30, 41, 44, 59
		Germany	3	30, 37, 68
		Italy	3	19, 20, 26
		Spain	3	6, 22, 51
		Finland	2	54, 55
		Norway	2	10, 16
		Sweden	2	28, 61
		Belgium	1	60
		Estonia	1	30
		Netherlands	1	30
		Romania	1	17
North America	16	USA	11	9, 15, 38, 40, 42, 43, 47, 50, 62, 65, 66
		Canada	5	8, 14, 33, 58, 64
Africa	12	South Africa	4	1, 2, 21, 52
		Nigeria	2	11, 39
		Burkina Faso	1	46
		Ethiopia	1	23
		Ghana	1	46
		Tanzania	1	46
		Uganda	1	5
		Zambia	1	67
Asia	9	Indonesia	2	32, 34
		Armenia	1	27
		Bangladesh	1	4
		China	1	57
		Israel	1	49
		Japan	1	53
		Pakistan	1	31
		Singapore	1	57
Australasia	7	Australia	7	7, 18, 25, 29, 36, 56, 63
South America	3	Brazil	2	24, 48
		Puerto Rico	1	35

The median year of publication was 2015 (Table 3).

**Table 3:** Year of publication for included papers in chronological order with corresponding study IDs. Multiple papers from the same study were included separately due to differing publication dates.

Year	Quantity	Study ID(s)
1992	1	43
2004	1	27
2005	1	31
2006	2	15, 25
2007	1	20
2008	2	16, 32
2010	4	1, 8, 14, 48
2011	1	59
2012	3	4, 18, 45
2013	5	8, 11, 32, 34, 45
2014	8	3, 11, 13, 35, 40, 41, 63, 64
2015	8	9, 19, 23, 38, 44, 47, 54, 60
2016	3	2, 21, 67
2017	3	12, 29, 5
2018	2	17, 61
2019	7	6, 24, 28, 39, 49, 50, 52
2020	7	10, 22, 30, 37, 58, 62, 65
2021	7	7, 26, 33, 36, 51, 56, 57
2022	2	34, 42

Most studies were qualitative, and all but two studies were observational, in that they presented evidence relating to existing uses of positive feedback (Table 4).

**Table 4:** Methods of included papers, in order of quantity. Multiple papers from the same study were counted as having a single study methods. Three companion papers were not counted in the ‘total quantity’ column. Many papers used multiple methods, each counted separately in the ‘quantity’ column.

Type of study	Total quantity	Method	Quantity
Qualitative	49	Interviews	40
		Focus groups	8
		Questionnaire/survey	4
		Observations	2
		Descriptive study	2
		Workshops	1
Quantitative	10	Questionnaire/survey	6
		Experience Sampling Method	1
		Observations	1
		Panel study	1
		Randomised study	1
Mixed	6	Interviews	5
		Questionnaire/survey	5
		Focus groups	3

The two intervention studies were as follows:

#### Riskin et al, 2019 [ID 49]

This study used pre-recorded video to simulate the impact on Neonatal Intensive Care Unit (NICU) team performance of gratitude expressed by two different sources. NICU teams (n = 43) were randomly assigned to 1 of 4 conditions: (1) maternal gratitude (2) physician-expressed gratitude (3) combined maternal and physician gratitude, or (4) control (same agents communicated neutral statements). Subsequent team performance in a training workshop was evaluated by a blinded panel, on a five-point Likert scale. Maternal gratitude produced a significant positive affect on team performance. Most of this effect was explained by the positive impact of gratitude on team information sharing. As a result, accuracy of diagnostic work was improved.

#### Weaver, 2020 [ID 65]

This study evaluated the impact of using a gamified feedback system on a medical-surgical unit in the US. The feedback system allowed service users to use a tablet to input free-text comments, which were later sent as text alerts to nurses and technicians. Its impact was evaluated using interviews, focus groups, and surveys. Healthcare staff described that receiving recognition and appreciation through the feedback system made them feel good, boosted confidence, morale and motivation, and helped them to feel comfortable in their job. Staff were initially enthusiastic about using the feedback system, which was seen to support the effect of positive feedback. Similarly, when staff became less enthusiastic and motivated to use the system over time, this hindered the effects of positive feedback. Night shift staff reported less opportunity to receive feedback from service users. The system was hindered by the lengthy process of accumulating points and rewards, making feedback from service users less timely, consistent, or meaningful.

### Objective 2—Characteristics of positive patient feedback in included studies

Positive feedback was described in included studies as having a variety of forms, most commonly described in their original papers as appreciation and gratitude (Table 5). The form of feedback was categorised as material or ambiguous. Material feedback referred to physical items given by service users, families, or the community. In a substantial number of included papers, the precise form of feedback was not explicitly stated, and hence has been identified in the table as ambiguous. For example, gratitude might be expressed through online systems or face-to-face interaction between healthcare staff and patients, but the form in which it was expressed was often not stated in published work, and instead papers talked more broadly about the impact of gratitude on healthcare staff.

**Table 5:** Positive feedback in included studies in order of quantity. Multiple papers from the same study were counted as having a single type of feedback.

Feedback category	Type of positive feedback	Quantity	Study ID(s)
Ambiguous	Appreciation	28	1, 2, 7, 8, 9, 11, 14, 19, 21, 24, 26, 29, 32, 33, 34, 36, 37, 43, 45, 47, 49, 51, 53, 54, 56, 61, 64, 68
	Gratitude	22	6, 10, 13, 14, 20, 21, 23, 24, 32, 33, 35, 42, 43, 46, 48, 52, 53, 57, 59, 60, 61, 62
	Thanks	16	5, 6, 14, 18, 30, 33, 35, 36, 40, 46, 47, 50, 54, 55, 65, 66
	Positive feedback	13	4, 15, 16, 17, 25, 28, 31, 38, 39, 40, 41, 44, 62
	Recognition	10	1, 11, 24, 27, 32, 43, 50, 55, 60, 68
	Praise	3	28, 41, 50
	Being valued	1	63
	Patient satisfaction	1	67
Material	Gifts	7	6, 14, 22, 25, 52, 58, 66
	Cards	5	14, 16, 18, 25, 65
	Flowers	2	52, 65
	Food	2	6, 62
	Hugs	1	16
	Letters	1	6

Included studies identified that positive feedback was delivered by service users (n = 53), the community (n = 18), and families (n = 16), with some studies identifying multiple sources of feedback.

Recipients of positive feedback were described using a broad variety of labels, most commonly identified as clinical staff providing direct care and treatment to service users (n = 68) (Table 6). In some studies, non-clinical staff received feedback (n = 3).

**Table 6:** Feedback recipients of positive feedback in included studies in order of quantity. Multiple papers from the same study were counted as a single feedback recipient.

Recipient category	Feedback recipient	Quantity	Study ID(s)
Clinical staff	Nurses	29	7, 8, 13, 16, 18, 19, 20, 24, 25, 26, 27, 28, 31, 32, 33, 37, 40, 42, 47, 48, 52, 53, 54, 55, 57, 60, 61, 62, 65
	Community health workers	7	4, 5, 7, 21, 23, 39, 67
	Physicians	6	14, 15, 43, 64, 66, 68
	Healthcare professionals	3	6, 22, 34
	Clinical staff	2	30, 58
	Frontline health workers	2	9, 56
	Health social workers	2	17, 43
	Healthcare personnel	2	10, 38
	Healthcare students	2	41, 51
	Midwives	2	27, 36
	Adult treatment team members	1	43
	Doctors	1	57
	Healthcare assistant	1	59
	Healthcare providers	1	45
	Healthcare workers	1	63
	Hospitalists	1	50
	Neonatal Intensive Care Unit team	1	49
	Nephrologists	1	44
	Primary health worker	1	11
	Psychologists	1	35
	Volunteer community caregivers	1	1
Non-clinical staff	Supervisors	1	2
	Technicians	1	65
	Disability support worker	1	29

Healthcare staff worked in a range of settings, categorised as clinical (primarily provides a health-related medical function) and non-clinical (primary purpose is not to provide a direct health-related medical function). Most studies considered clinical settings (n = 74) (Table 7). Two included papers did not explicitly state the setting [49, 50].

**Table 7:** Feedback settings of positive feedback delivery in included studies in order of quantity. Multiple papers from the same study were counted separately only if the setting differed between papers.

Setting category	Feedback setting	Quantity	Study ID(s)
Clinical setting	Hospitals	27	8, 16, 17, 19, 20, 22, 25, 26, 28, 31, 35, 36, 37, 40, 41, 42, 49, 50, 51, 52, 54, 56, 57, 60, 62, 65, 68
	Oncology	6	8, 19, 30, 41, 43, 47
	Emergency department	4	13, 17, 19, 31
	Clinics	3	16, 32, 38
	General Practice (GP)	3	7, 25, 59
	Health centres	3	18, 45, 58
	Intensive care	3	28, 49, 52
	Maternal care	3	17, 25, 31
	Medical surgery	3	31, 47, 65
	Nursing homes	3	10, 41, 51
	Primary care	3	11, 15, 34
	Ambulatory care	2	8, 31
	Hospices	2	17, 41
	Covid-19 unit	1	51
	Critical care	1	31
	Family health units	1	24
	Palliative care	1	6
	Private practice	1	35
	Renal department	1	44
	Reproductive health services	1	27
	Sexual violence services	1	48
	Transplant coordination	1	22
Non-clinical setting	Community (including home-based care and faith-based organisations)	19	1, 2, 4, 5, 6, 7, 8, 9, 12, 14, 17, 21, 23, 26, 39, 41, 53, 64, 67
	University	3	28, 35, 66
	Disability services	1	29
	School-based	1	17
	Telephone services	1	61

### Objective 3: Measured used to quantify change

There was a considerable variation in the outcome domains and measures used in studies (n = 11) (Table 8). The remaining 57 studies did not include a standardised outcome measure. A measure was concluded to be standardised if a citable reference was available.

**Table 8:** Outcome domains and outcome measures used in included studies.

Outcome domain	Standardised outcome measure	Quantity	Study ID(s)
Attitudes towards caring for dying patients	Frommelt Attitudes Toward Care of the Dying scale, form B, Japanese version (FATCOD B-J)	1	53
Attitudes towards death	The Death Attitude Inventory (DAJ)	1	53
Baseline affective states	Short Positive and Negative Affect Schedule (PANAS)	1	57
Beliefs, attitudes, experiences of gifts	Scale of Attitudes and Behaviors toward Gifts in Psychotherapy (SABGP)	1	35
Burnout	Maslach Burnout Inventory	2	50, 68
Burnout	Maslach Burnout Inventory for Human Service Sector	1	19
Completion of clinical/non-clinical tasks	MEASURE Evaluation’s Quick Investigation of Quality (QIQ) tool	1	27
Engagement at work	Gallup Worker Engagement Survey	2	50, 65
Experiences, understandings, meanings	Nursing Practice in Rural and Remote Canada II (RRNII)	1	33
Illegitimate tasks	Bern Illegitimate Tasks Scale	1	37
Job satisfaction	Job Enjoyment Scale	1	65
Patient behaviour as a psychological resource	Customer-initiated support scale	1	19
Patient satisfaction	Hospital Consumer Assessment of Health Plans Survey (HCAHPS)	1	65
Perception of service user gratitude	PGRate scale	1	19
Psychological demands	Job Content Questionnaire (JCQ) subscales	1	19
Resilience	Abbreviated 2 item Connor-Davidson Resilience Scale (CD-RISC 2)	1	56
Resilience	Abbreviated Impact of Event Scale (IES-6)	1	56
Resilience	Abbreviated Maslach Burnout Inventory (AMBI)	1	56
Resilience	Patient Health Questionnaire (PHQ-9)	1	56
Resilience	The Generalised Anxiety Disorder (GAD-7)	1	56

### Objective 4: Types of change, and how it occurs

#### Outcomes

All identified outcomes were reported as change for healthcare staff, rather than a change to a healthcare system. Three papers reported a change in the therapeutic staff-service user relationship rather than the healthcare staff individually. Outcomes reporting a change in staff-service user relationships describe a strengthened therapeutic alliance [51–53]. Outcomes categorised as helpful are described in Table 9.

**Table 9:** Helpful outcomes identified in included studies, arranged by higher-level category and sub-category. Multiple papers from the same study were counted separately only if reporting different outcomes. Some outcomes were described ambiguously in their original papers and therefore included in, but not expanded on, in the table.

Higher category	Outcomes	Study ID(s)
Short-term emotional change for healthcare workers	Boosted confidence	40, 41, 65
	Boosted morale	38, 65
	Confirmation of doing good work	16, 18, 25, 28, 33, 41, 42, 50, 58, 62
	Coping resource at work	55
	Enthusiasm for the job	54
	Experience of having a good day	50, 60
	Feeling comfortable in their job	65
	Feeling empowered	7
	Feeling encouraged	5, 11, 28, 42, 45, 54, 55
	Feeling engaged	50
	Feeling fulfilled	6, 39
	Feeling good	38, 40, 41, 48, 51, 65
	Feeling happy	10, 24, 29, 38, 39, 55
	Feeling honoured to serve their community	33
	Feeling inspired	1, 54
	Feeling positive about work	51, 53, 63
	Feeling proud of work	2, 6
	Feeling rewarded	1, 6, 20, 24, 29, 40, 43, 44, 45, 54, 55, 59, 62
	Feeling successful	10
	Feeling supported	7
	Feeling valued	2, 7, 36, 55, 58, 63
	Feelings of hope	26
	Feelings of love for work	30
	Feeling that the reciprocal respect between service user and healthcare worker is fulfilled	52
	Increased individual energy at work	30, 58
	Increased gratification	22, 33, 48, 68
	Increased gratitude of healthcare workers	6, 55
	Increased motivation at work	2, 5, 6, 11, 21, 23, 24, 27, 28, 30, 32, 37, 38, 39, 42, 46, 47, 54, 55, 65, 67
	Increased personal satisfaction	51, 58
	Increased psychological wellbeing	6, 7, 36, 56, 58
	Increased sense of achievement	45
	Greater self-reflection about practice	6
	Source of strength/support during difficult times	6, 28, 68
Work-home interactional change for healthcare workers	Improved familial satisfaction for spouses of healthcare workers	57
	Improved work-home relationship	57
Work-related change for healthcare workers	Created a positive work environment	61
	Improved communication with service users	32
	Improved team diagnostic and procedural performance	49
	Increased commitment to work	28, 31, 54, 55
	Increased connection to service users and families	50, 68
	Increased intention to refer to a service being positively evaluated	15
	Increased sense of doing meaningful work	16, 24, 45, 50
	Increased staff retention	3, 6, 8, 9, 14, 38, 63, 64
	Increased work-related activity	4
	Increased work-related satisfaction	1, 6, 9, 13, 17, 20, 23, 24, 28, 31, 32, 33, 34, 38, 43, 46, 48, 50, 51, 54, 55, 58, 59, 61, 62, 63
	Reduced burnout	6, 19, 56
	Reduced perception that assigned tasks are avoidable or outside of job role responsibility	37
	Strengthened therapeutic alliance	35

Some papers identified undesirable changes (Table 10).

**Table 10:** Undesirable changes for healthcare staff identified in included studies.

Change category	Sub-category	Study ID(s)
Short-term emotional change for healthcare workers	Feeling embarrassed when being delivered feedback from tutors	41
	Feelings of envy and stress when not rewarded with positive feedback	55
	Feelings of guilt after accepting a gift	35
	Feelings of tension and pressure to meet philanthropic service user expectations	66

One change was identified which could be viewed as both helpful and undesirable depending upon the healthcare context. An altered responsiveness to grateful service users who give philanthropic gifts could be viewed as helpful in a healthcare system that values donations, as responding more quickly to those giving gifts may increase the likelihood of future donations [52]. However, altered responsiveness may undermine the professional relationship between staff and service-users and result in a decreased responsiveness to those not giving gifts.

#### Mechanisms

A mechanism is a process by which positive feedback causes change. Mechanisms identified in included studies are in Table 11.

**Table 11:** Mechanisms identified as cause of change in included studies.

Mechanism	Study ID(s)
Construction of professional identity	17
Reflection on practice	41
Intensified prosocial behaviour	49
Protective resource against secondary trauma	36
Relationship shift between staff and service user [after gift-giving]	66
Validation [of role and performance]	8, 17, 30

#### Moderators, facilitators, and barriers

Factors were identified which can alter the degree of change following positive feedback. Some factors enhanced the effect of positive feedback (Table 12).

**Table 12:** Factors enhancing the effect of positive feedback in included studies.

Higher-category factors enhancing change	Specific factors enhancing change	Study ID(s)
Healthcare role has characteristics enabling change	Staff work in the oncology department	19, 43, 47
	Psychological demands of healthcare role are manageable	19
Healthcare staff have characteristics enabling change	Staff are enthusiastic about feedback system	65
	Staff are confident when asking for feedback	41
	Staff perceive events positively	68
	Staff have previous experience of working in an environment focussing on negative feedback	41
	Staff have strong occupational identity	57
	Staff value service users as the source of positive feedback	37, 49
	Staff are confident using Personal Protective Equipment	56
Feedback has characteristics enabling change	Positive feedback is received frequently	6
	Feedback given is genuine and central to staff identity	49

Some studies also identified barriers to change, where the effect of positive feedback was hindered (Table 13).

**Table 13:** Factors hindering the effect of positive feedback in included studies.

Higher-category factors hindering change	Specific factors hindering change	Study ID(s)
Healthcare role has characteristics hindering change	Staff receive positive feedback as a result of other absent medical staff who have delegated tasks; dissatisfaction overshadows positive effect of feedback	20
	Being a nurse compared to being a doctor associated with reduced positive beliefs about community appreciation	56
	Staff experience negative stigma faced during the Covid-19 pandemic as ’plague spreaders’	26
	Staff have less opportunity to gain feedback (e.g., night-shift staff)	65
	Staff work in the medical-surgical department	47
Healthcare staff have characteristics hindering change	Staff experience confidence issues when requesting feedback from service users	41
	Staff are not enthusiastic about feedback system	65
	Staff feel burdensome when asking for feedback from those who have received bad news	41
Feedback system hinders change	Feedback system is time-consuming	65
	Feedback is not consistently given	32

Some studies described characteristics of specific healthcare roles that enhanced the impact of positive feedback. Three studies described working in oncology as enhancing the effects of positive feedback. One study described having increased intimacy and closeness with oncology service users, facilitating feelings of reward and satisfaction [54]. Another described how working in oncology felt more worthwhile and like a gift, with service users expressing deep appreciation which is not seen in other wards.

One study described how working in oncology had fewer psychological demands [55]. The psychological demands of the healthcare role impacted the degree of change between service user gratitude and burnout. Emergency units were perceived to have higher psychological demands than oncology wards, due to work shifts, workloads, and the shorter, more superficial relationships with service users. For emergency nurses, personal accomplishment as a mediator of burnout diminished with increased psychological demands. In contrast, oncology nurses had higher perceptions of service user gratitude and higher personal accomplishment. The institutional context may influence the extent to which staff members are able to encounter and engage with positive feedback.

Occupational identity was also identified in another study as factor enhancing the effect of service user gratitude, with changes to energy within relationships, spousal family satisfaction, and relationship-based family performance [56]. Receiving service user gratitude improved healthcare staff’s home environment, and this was amplified when staff strongly identified with their role.

In one study, appreciation reduced the relationship between intrinsic motivation (a type of motivation that is based on inherent pleasure or passion, rather than extrinsic rewards such as money or fame) and the perception of illegitimate tasks [57]. Illegitimate tasks were unnecessary (tasks that could have been avoided with better organisation) or unreasonable (tasks that were not the responsibility of that staff member). Motivated staff perceived a higher number of unnecessary tasks being assigned to them, but appreciation from service users reduced this relationship.

#### Mediators

A mediator is a factor which is essential in the change process and must be in place for change to occur. In the study by Riskin et al (2019), team information sharing partially mediated the impact of gratitude [58]. In a study by Tang et al (2021) energy within relationships mediated the effect of service user gratitude and spousal family satisfaction and relationship-based family role performance [56]. Receiving gratitude from service users acts as an energy resource within relationships, which healthcare staff are then able to utilise in the family domain. As a result, increased relational energy led to increased familial satisfaction.

### Subgroup analyses

#### Quality assessment

Only one study (reported on in two papers) did not meet the 60% threshold for quality assessment due to a lack of a clear research question [46, 59]. Findings from this study were not consequential to the change model due to these being reinforced by other studies [46].

#### Studies conducted in a mostly public versus mostly private healthcare system

One difference between studies conducted in a mostly public healthcare system (UK) and mostly private healthcare systems (US) was the type of positive feedback provided. All UK studies described ambiguous types of positive feedback. While many US studies also described ambiguous feedback, two described material feedback in the form of cards, flowers, and gifts [52, 60]. One undesirable change was identified in both UK and US studies. In the UK, research identified that students feel embarrassed when receiving positive feedback from feedback forms via tutors [61], whereas in the US, tension and pressure surrounding the service user-professional relationship was identified after gift-giving [52].

## Discussion

### Summary of findings

The review included a broad range of papers presenting evidence that change can be created in health services using positive patient feedback. The largest body of evidence relates to beneficial short-term emotional changes experienced by healthcare workers as the result of receiving feedback, such as feeling more hopeful and motivated, and to beneficial work-related change (such as increased retention and reduced burnout). Beneficial changes to the home environment were also documented. A small number of undesirable changes were identified. These included feeling embarrassed when receiving feedback, feeling envy and stress when not rewarded with positive feedback, and feeling guilt, tension, and pressure when accepting gifts. Tensions surrounding service user gift-giving may arise due to health professionals being restricted to only accepting ‘trivial’ gifts, which may create uncertainty in staff regarding boundaries due to vague definitions [62]. The type of gift (such as those marking an occasion, inexpensive, or ‘over the top’) and recipient (such as individual staff or donation to the service) may influence staff reactions. Gifts which fail to align with ethical practice, such as ‘over the top’ displays of gratitude, may be more likely to produce undesirable change [63].

Importantly, only two intervention studies were identified [58, 60], and neither quantified effect in a real-world healthcare setting. This means that no evidence on the size of effect produced by positive feedback was available. This points to a substantial gap in knowledge which might be addressed by future research studies. A broad range of measures were used in quantitative studies, suggesting a lack of consensus in the research community on the most important constructs to consider, and how to assess them. Most work has been conducted within the last 10 years, which potentially relates to the widespread emergence of technological solutions to the collection and distribution of feedback, creating the potential for new forms of intervention.

The current review has identified factors which enhance or hinder the creation of change through feedback. Some of these factors relate directly to the nature of specific healthcare roles and professions. For example, change was enhanced if feedback recipients worked in roles which allow more meaningful interaction with service users, and hindered for feedback recipients working night shifts and hence potentially having less direct contact with patients. This suggests that positive feedback may not be an accurate measure for assessing quality of care as some staff are not given the opportunity to influence and receive feedback. It is unlikely that feedback will be equally received by staff across services due to their varying nature with the implementation of a single feedback system. Tailoring feedback systems to the settings and contexts in which staff work may be beneficial to ensure similar opportunities to receive feedback but understanding the fundamental differences between services is crucial when assessing quality improvement priorities.

### Relationship to prior work

The current review extends a previous systematised review which investigated how expressions of service user gratitude creates change in healthcare services [29]. Due to the current review having a mostly broader focus, 68 papers were included compared to 26 papers in the previous review, and this has resulted in a broader range of short-term emotional benefits and undesirable impacts being identified.

In a scoping review investigating service user gratitude in healthcare, receiving gratitude was found to enhance healthcare worker wellbeing, act as a positive force against stress, increase motivation, increase reciprocated gratitude, and reduce burnout [27]. Aparicio and colleagues identified 32 includable papers, only two of which were included in the current review [55, 64]. Despite a lack of cross-over in included studies due to differences in inclusion criteria, the findings remain consistent. For instance, gratitude acting as a positive force against distress is also seen in the current review, categorised as increased psychological wellbeing and a protective force against trauma.

The benefits of positive feedback identified in this review may be particularly relevant for the occupational health of healthcare staff. For example, in the UK, the number of nurses leaving the profession rose in 2021 by 25% [65], with increased workload leading to higher levels of burnout [66]. Healthcare workers have been found to have high levels of intrinsic motivation, where motivation to perform well is a product of inner drives. This was particularly evident in permanent healthcare staff [67]. Validation of having done good work may therefore be positively reinforced with positive feedback and be of greater value than for those who are extrinsically motivated by factors such as financial reward or promotion [68]. Increased intrinsic motivation may boost affective commitment and lead to reduced turnover intention among healthcare staff [69]. Similarly, finding intrinsic meaning in their work was helpful for healthcare workers in Japan to cope during the COVID-19 pandemic [25]. Self-determination theory also suggests that intrinsic motivation can assist with the development of professional identity for healthcare staff [70].

The current review has identified that characteristics of healthcare staff can influence the change created by positive feedback. Many relevant characteristics will be modifiable (such as enthusiasm about feedback systems), and interventions to shape healthcare staff attitudes surrounding service user feedback may be essential for implementing meaningful change, for example due to a widespread belief that feedback is largely negative [18]. The Lewin Change model describes three steps for creating change [71], starting with ‘unfreezing’ whereby a shift away from current beliefs is initiated through challenging defensiveness towards change and dismantling current views. This may be possible through exposure to positive feedback. The second stage is ‘movement’ which describes a change occurring, such as beneficial outcomes as a result of positive feedback. The third stage is ‘refreezing’ which describes a replacement of old views and processes with new ones, which begins to normalise the new methods of operating. For positive feedback in healthcare, this may reflect system-level change such as policy implementation.

However, this model may be limited to healthcare staff’s willingness to engage with positive feedback. The idea of a ‘learning organisation’ was introduced by Senge, who described a group of people continually working to enhance their capacities and create results that they want [72]. A learning organisation describes one which is not operating as a machine, but rather a humanistic never-ending process of development and learning. Adapted for healthcare settings, learning organisations have five disciplines [73]. ‘Open systems thinking’ describes services being viewed as a whole rather than isolated by disease, procedures, or structures, and aims to create interconnectedness beyond departmental boundaries. ‘Improving individual capabilities’ describes striving for excellence by improving personal proficiencies of staff. ‘Team learning’ describes learning as a collective rather than via single professionals. ‘Updating mental models’ describes updating the deeply held assumptions and generalisations held by individuals within the organisation and finding new ways of operating. Finally, ‘a cohesive vision’ describes empowering and enabling staff being counterbalanced by strategic direction and clear values to guide individual action to produce shared understanding. Healthcare systems have identified that being a ‘learning organisation’ encourages a culture celebrating innovation and success [73]. Positive feedback may offer a means for learning organisations to create a cultural shift towards valuing positive service user experiences rather than focussing solely on negative incidents and risk reduction.

### Strengths and limitations

A strength of the review is that a broad range of publications databases was searched, including a database specific to computing publications and rarely used in health-related reviews, which is important when feedback is routinely collected through technological means. Compared to the prior narrower review, broader inclusion criteria have enabled the inclusion of papers describing changes to healthcare systems, enabling the identification of changes such as increased referral intentions following positive feedback from service users about a particular service [74]. The addition of search terms such as ‘positive feedback’ and ‘positive evaluation’ have enabled new forms of change to be identified, such as non-clinical staff benefiting from positive feedback as well as those in clinical roles. Inclusion criteria were carefully designed to exclude papers where there was ambiguity about the source of feedback or the direction of change, meaning that studies were excluded where causality was uncertain, such as in studies using correlation analyses [75]. This has provided a solid foundation to develop a change model.

Another strength of the review is that it was inclusive of studies which were conducted in non-WEIRD (western, educated, industrialised, rich, and democratic) countries. For example, included studies reflected healthcare systems in eight African regions. Although emotional expressions differ across cultures [76], positive feedback was deemed helpful to healthcare organisational outcomes. Findings were robust across studies despite differing locations and healthcare systems, reinforcing the value of positive feedback. Expanding the review to include papers not published in English would strengthen findings.

A limitation of the review is that the definition of positive feedback is not straightforward. A subgroup analysis was planned for documents which identify change through expressions of healthcare service user gratitude specifically. Ambiguity in the distinction between positive feedback and gratitude definitions meant that the subgroup analysis could not be performed. Medical definitions of positive feedback describe the body being amplified from its normal state [77], but this review did not include positive physical or medical signals from service users. However, seeing a patient improve was described in some studies as a form of positive feedback [78]. Physiological markers may not reflect positive healthcare experiences and would not reflect quality of care given by palliative care teams. Further, service user gratitude was seen to create change for other service users [26], but this was excluded as it could not be considered a change for healthcare staff or systems.

Positive feedback was defined as a response from healthcare service users, families or the community indicating concordance between desired and actual experiences regarding their care or treatment, delivered to healthcare staff or systems. However, the assumption was made that positive feedback was expressed with the intention of communicating this concordance between desired and actual care, but other contextual and motivating factors may have existed, such as feeling obligated to give positive responses when asked for feedback in person [79], service users attempting to influence their future care and treatment and prevent punitive treatment for negative feedback [26], and social norms surrounding expressions of thanks which may be expressed habitually [80].

In seeking to provide a broad summary of existing knowledge, the review has used broad change modelling concepts such as moderation and mediation to synthesise findings from potentially disparate studies. A limitation of this approach to synthesis is that it does not provide a route to documenting rich contextual detail needed to understand how change occurs in specific settings. This approach to synthesis has to potential to overemphasise causality, for example through propagating an overemphasis of causality present in included papers.

### Implications of the review and change model

#### Implications for practice

Managers of health service units seeking to address problems such as staff burnout or low motivation should consider the integration of mechanisms for making positive feedback available to staff members and should seek to identify barriers to the use of positive feedback in their units. Health service managers in units already making use of positive feedback should examine whether particular staff groups are disadvantaged, for example if working in circumstances that make the provision of positive feedback more difficult, or increasing exposure of positive feedback to individuals from minority ethnic backgrounds who may be more likely to receive complaints [81]. Policymakers should consider adopting policies that encourage the collection and distribution of positive feedback. Requirements of healthcare professional bodies to make use of feedback in reflective practice might be used to motivate change, though it is unclear whether this phenomenon extends beyond the UK. This may also exclude individuals whose roles do not require professional registration. Integrating positive feedback from service users, families, or communities into standard clinical supervision rather than formal requirements may create an attitudinal shift away from revalidation scepticism to become an essential part of practice [17]. Effective clinical supervision can prevent burnout [82], and positive feedback may enhance these benefits.

#### Implications for research

Only two interventional studies were included in the review, which limits knowledge on the scale of effect of positive feedback. Researchers should consider developing interventions incorporating positive feedback, and evaluating their use in real world settings. The research community should seek to reach consensus on the most important measures to be assessed interventional studies to enable meta-analyses work. Future research may investigate the effects of positive feedback depending on healthcare role, comparing those who have consistent access to feedback (such as oncology staff) [64], to those who feel overlooked and undervalued (such as healthcare assistants) [83]. Future research may investigate the effects of positive feedback at multiple levels of the organisation, such as individual impacts like resilience, and organisational culture and system-level change, and whether the effect of positive feedback changes depending on individual or team receipt.

The research community should also aim to investigate the influence of feedback content and form in eliciting change and whether content has practical utility. Examples include whether content of feedback is meaningful to staff, and if relationships with service users are more significant than numerical indicators of satisfaction. Feedback with specific utility, such as an appointment being ‘on time’, may also produce differing effects to interpersonal emotional connections. This may assist with the development of a typology to characterise feedback and assist with understanding whether positive feedback should be used and delivered universally.

Research may also benefit from being co-designed with healthcare workers with practical knowledge to enhance the functional integration of findings into clinical practice.

## Conclusions

As described in the current empirical research literature, change created by positive feedback is largely positive, with emotional, familial, and work-related change being recognised. Some undesirable changes were identified in relation to healthcare staff emotions. Insufficient interventional research has been conducted to establish whether positive feedback is effective or cost-effectiveness at creating specific forms of change, and hence such research should be a priority for the research community. Healthcare managers may wish to use positive feedback more regularly, and to address barriers to staff receiving feedback.

## Supporting information

S1 File Amendments to search strategy for CINAHL, ASSIA and the ACM digital library. (PDF)

S2 File PRISMA checklist. (PDF)

S1 Table Full data abstraction table. (XLSX)
