# Supplementary material for: Health service improvement using positive patient feedback: Systematic scoping review
Source: PLoS One. 2023 Oct 5;18(10):e0275045. doi: 10.1371/journal.pone.0275045 (PMC10553339; doi:10.1371/journal.pone.0275045)
Supplement: S1 File — (PDF) [file pone.0275045.s001.pdf]

Amended search strategy for CINAHL and ASSIA

1. TI “health* staff” or AB “health* staff”
2. TI “health* worker*” or AB “health* worker*”
3. TI “medical staff” or AB “medical staff”
4. TI “medical worker*” or AB “medical worker*”
5. MW Health Personnel
6. TI “health* system*” or AB “health* system”
7. TI “health* service*” or AB “health* service*”
8. TI “health* organi#ation*” or AB “health* organi#ation*”
9. TI “health* communit*” or AB “health* communit*”
10. (S1 OR S2 OR S3 OR S4 OR S5 OR S6 OR S7 OR S8 or S9)
11. TI “grat*” or AB “grat*”
12. TI “appreciat*” or AB “appreciat*”
13. TI “recog*”
14. TI “thank*”
15. TI “positive* feedback” or AB “positive* feedback”
16. TI “positive* evaluat*” or AB “positive* evaluat*”
17. TI “praise*” or AB “praise*”
18. (S11 OR S12 OR S13 OR S14 OR S15 OR S16 OR S17)
19. (S10 AND S18)

Searches conducted on the ACM Digital Library

1. Health* staff AND grat*
2. Health* staff AND appreciat*
3. Health* staff AND positive* feedback
4. Health* staff AND positive evaluat*
5. Health* worker* AND grat*
6. Health* worker* AND appreciat*
7. Health* worker* AND positive* feedback
8. Health* worker* AND positive* evaluat*
9. Health* system* AND grat*
10. Health* system* AND appreciat*
11. Health* system* AND positive* feedback
12. Health* system* AND positive* evaluat*
13. Health* service* AND grat*
14. Health* service* AND appreciat*
15. Health* service* AND positive* feedback
16. Health* service* AND positive* evaluat*

This work is licensed under the Creative Commons Attribution 4.0 International License. To view a copy of this license, visit http://creativecommons.org/licenses/by/4.0/ or send a letter to Creative Commons, PO Box 1866, Mountain View, CA 94042, USA. To attribute the authors, cite as: Lloyd R and Rennick-Egglestone S. Search strategies for a systematic review on positive patient feedback (2022). Include the URL where the file was published in this citation.
